# Toxicology and clinical potential of nanoparticles

**DOI:** 10.1016/j.nantod.2011.10.001

**Published:** 2011-12

**Authors:** Lara Yildirimer, Nguyen T.K. Thanh, Marilena Loizidou, Alexander M. Seifalian

**Affiliations:** a Centre for Nanotechnology & Regenerative Medicine, UCL Division of Surgery & Interventional Science, University College London, London, UK; b Department of Physics and Astronomy, University College London, Gower Street, London WC1E 6BT, UK; c The Davy Faraday Research Laboratory, The Royal Institution of Great Britain, 21 Albemarle Street, London W1S 4BS, UK; d Royal Free Hampstead NHS Trust Hospital, London, UK

**Keywords:** Nanoparticles, Toxicity, Polymeric, Carbon nanotubes, Silica, Silver, Gold, Quantum dots, Superparamagnetic iron oxide, Route of administration, Administered dose, Pulmonary, Oral, Transdermal, Intravenous, Lung, Skin

## Abstract

In recent years, nanoparticles (NPs) have increasingly found practical applications in technology, research and medicine. The small particle size coupled to their unique chemical and physical properties is thought to underlie their exploitable biomedical activities. Here, we review current toxicity studies of NPs with clinical potential. Mechanisms of cytotoxicity are discussed and the problem of extrapolating knowledge gained from cell-based studies into a human scenario is highlighted. The so-called ‘proof-of-principle’ approach, whereby ultra-high NP concentrations are used to ensure cytotoxicity, is evaluated on the basis of two considerations; firstly, from a scientific perspective, the concentrations used are in no way related to the actual doses required which, in many instances, discourages further vital investigations. Secondly, these inaccurate results cast doubt on the science of nanomedicine and thus, quite dangerously, encourage unnecessary alarm in the public. In this context, the discrepancies between *in vitro* and *in vivo* results are described along with the need for a unifying protocol for reliable and realistic toxicity reports.

## Graphical abstract

**Graphical abstract:**

**Figure:**
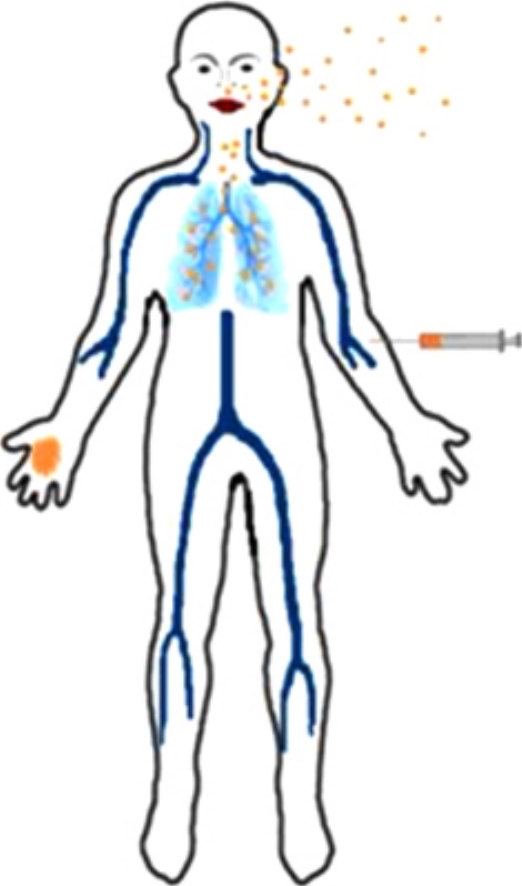

**Highlights:**

▶ NP toxicity depends on NP characteristics, administered dose and route. ▶ *In vitro* toxicity results do not easily translate into *in vivo* toxicity. ▶ Current research lacks a unifying protocol for the toxicological profiling of NPs.

## Introduction

Nanoparticles have a large surface area to volume ratio which leads to an alteration in biological activity compared to the parent bulk materials. In the past two decades, the use of nanoparticles (NPs) in experimental and clinical settings has risen exponentially due to their wide range of biomedical applications, for example in drug delivery, imaging and cell tracking [1–4]. This highlights the need to consider not only the usefulness of NPs but also the potentially unpredictable and adverse consequences of human exposure thereto. In this context, NP toxicity refers to the ability of the particles to adversely affect the normal physiology as well as to directly interrupt the normal structure of organs and tissues of humans and animals. It is widely accepted that toxicity depends on physiochemical parameters such as particle size, shape, surface charge and chemistry, composition, and subsequent NPs stability. The exact underlying mechanism is as yet unknown, however, recent literature suggests cytotoxicity to be related to oxidative stress and pro-inflammatory gene activation [5–7]. Further to particle-related factors, the administered dose, route of administration and extent of tissue distribution seem important parameters in nano-cytotoxicity. Typically, cell-based toxicity studies use increasing doses of the NP in order to observe dose-related cellular or tissular toxicity. Such dose–response correlations are the basis for determining safe limits of particle concentrations for *in vivo* administration. Despite the theoretically brilliant logic, animal and human studies have taught us differently and highlighted the issue of the feasibility of correlating organ toxicity with the pre-determined dose; there exists a widely acknowledged problem of extrapolating *in vitro* concentrations into *in vivo* scenarios which can be subdivided into two points; firstly, it has yet to be determined how efficiently any administered NP dose is reaching the target tissue and secondly, NPs can induce biochemical changes *in vivo* which may have gone unnoticed in isolated cell-based studies. With the potentially disastrous consequences in mind, new ways of predicting as yet unpredictable, non-dosage-dependent actions of NPs *in vivo* must be sought. Apart from the dosing issue, another, so far underexposed area of nanotoxicity relates to the route of particle administration which may also, quite independently from the dose, influence toxicity in an adverse fashion. It is sensible to assume that biodistribution, accumulation, metabolism and excretion of NPs will differ depending on the route of administration as will its toxicity. So far, no reviews have focused on the association between different routes of administration and NP toxicity.

**Figure 1:**
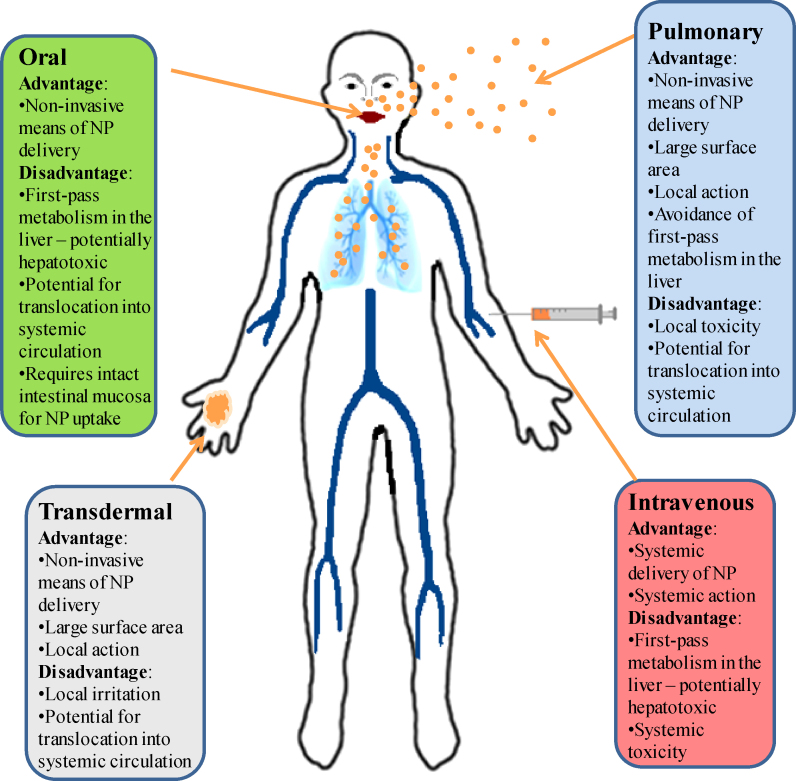
Routes of administration of nanoparticles and their advantages and disadvantages.

Substances may enter the body via oral ingestion, inhalation, dermal penetration and intravascular injection and subsequently distribute to any organ system. Fig. 1 summarizes the advantages and disadvantages of each of the routes. Pulmonary drug delivery shows tremendous potential but concerns regarding local and systemic toxicity currently curb enthusiasm [8]. NP aggregation and subsequent tissue inflammatory reactions have been postulated to be the underlying mechanism [9–11]. Topically applicable substances such as sunscreen preparations and cosmetics already rely on the use of nano-formulations of titanium- and zinc-dioxide by exploiting their ultraviolet radiation blocking ability. In the future, the penetrative capacity of certain NPs could be exploited for transdermal drug delivery. Therefore, the mechanistics of transition and potential dermal or systemic toxicity need to be evaluated. Intravenous and oral NP administrations inherently have a more rapid systemic effect compared to transdermal administration and once within the circulation, most substances are subject to first-pass metabolism within the liver where they may accumulate or distribute via the vasculature to end organs including the brain. Despite its innate protection by the blood–brain-barrier (BBB) against external chemical insults, the potential for nanoparticulate matter to percolate through tight junctions renders the brain vulnerable to potential particle-mediated toxicity. Reliable data on NP toxicity is therefore necessary to avoid detrimental adverse effects.

In this review, we aim to identify clinically relevant NPs and critically appraise organ-based toxicological studies which have been carried out on a cellular and pre-clinical level as well as on human volunteers. Furthermore, emphasis will be placed upon the importance of particle size and the route of administration with respect to toxicity as the authors believe that the latter has not received adequate evaluation despite its fundamental importance with respect to the clinical setting.

## Types of nanoparticles for clinical applications

NPs have a vast potential in the medical arena as drug and gene delivery vehicles, fluorescent labels and contrast agents [1,12]. For a particle to qualify as a “true” NP, at least one of its material dimensions must lie within the size range of 1–100 nm. The use of NPs as carrier systems for drugs, particularly chemotherapeutic drugs, is gaining in popularity due to the ability to specifically target cancer cells, enhance efficacy and reduce systemic toxicity. Gold NPs (AuNP) show several advantageous properties including a non-toxic and biocompatible metal core making them an ideal starting point for nanocarrier systems [13]. Furthermore, AuNP can undergo multiple surface functionalizations combining different moieties such as drugs and targeting agents which renders them a highly versatile tool for targeting [14] (Fig. 3). Other drug nano-vehicles which are already in clinical use include lipid-based [15], polymer-based [16] and biological NPs [17]. Quantum dots (QDs), or semiconductor nanocrystals, are another type of NPs which enjoy a wide range of potential clinical applications including cell labeling, *in vivo* imaging and diagnostics [18]. Due to the QD's exceptional photophysical properties such as broad absorption spectra coupled to a narrow emission spectrum, QDs of different emission colors may be simultaneously excited by a single wavelength, thus enabling multiplexed detection of molecular targets [19]. Other fluorescent labels used in medical research include magnetic NPs such as superparamagnetic iron oxide nanoparticles (SPIONs) [20]. SPIONs are one of the few clinically approved metal oxide NPs and find ubiquitous applications in the biomedical field such as magnetic resonance imaging (MRI) [21], drug [22] and gene delivery [23] and hyperthermic destruction of tumor tissue [22]. Their superparamagnetism confers several advantages: firstly, the ability to be guided by means of an external magnetic field may be exploited in targeted imaging or drug delivery systems; secondly, the production of cytotoxic heat when subjected to alternating magnetic fields could be utilized in cancer treatments [24]. A different modality of exposure to metal oxide NPs comprises that of topically applicable formulations such as creams and sunscreen lotions containing titanium dioxide and zinc oxide NPs [25]. Here, it is important to determine whether NPs can penetrate deeper into skin layers and possibly be absorbed into the systemic circulation and accumulate in tissues. Nanoscaled silver (AgNP) became popular for its marked antimicrobial effect which is successfully exploited in medical applications such as silver-impregnated wound dressings, contraceptive devices, surgical instruments and bone prostheses [26–29]. Carbon nanotubes are made from rolled up sheets of graphene and are classified as single-walled (SWCNTs) or multi-walled carbon nanotubes (MWCNTs) depending on the constituent numbers of graphene layers. Due to their unique size and shape, much effort has been dedicated to analyzing biomedical applications of CNTs. Such extensive potential requires the meticulous evaluation of toxicity.

**Figure 2:**
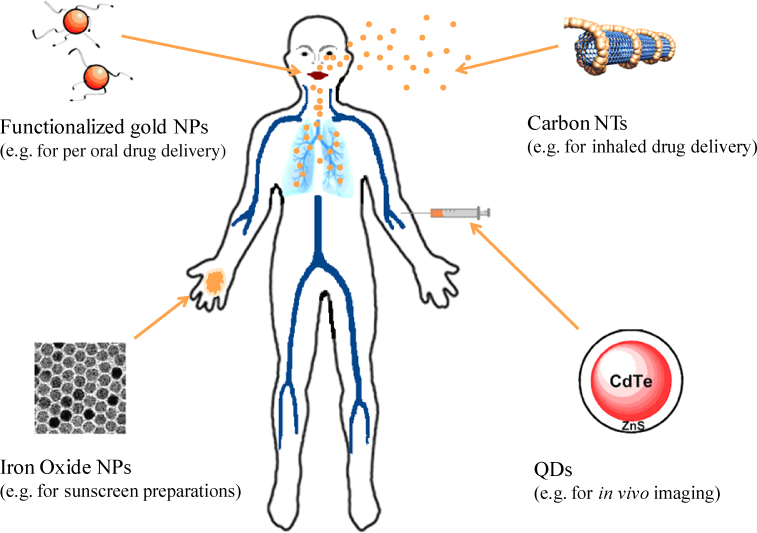
Selection of biologically useful nanoparticles [30].

**Figure 3:**
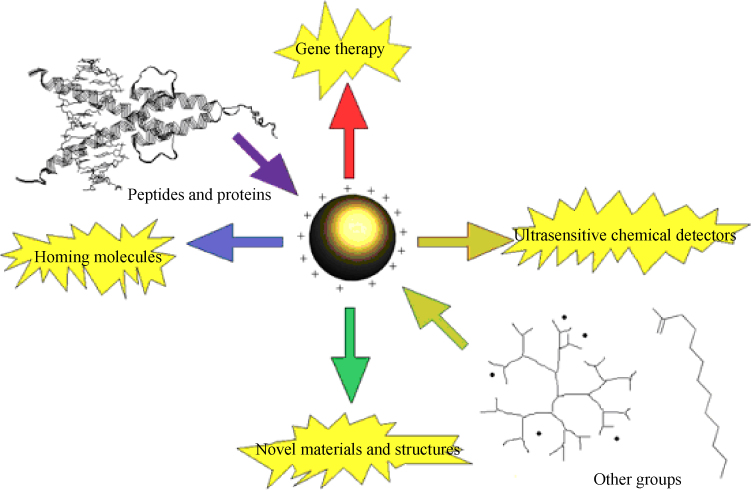
Schematic representation of functionalization potential of gold nanoparticles [120].

This widespread use of different types of NPs in the biomedical field raises concerns over their increasing access to tissues and organs of the human body and, consequently, the potential toxic effects. Various studies evaluating *in vitro* and *in vivo* absorption, distribution and biocompatibility of NPs have been reviewed and are critically appraised in this article. Fig. 2 shows a summary of biologically important nanoparticles and their possible routes of administration (adapted from reference [30]).

## Toxicological profiling in cell based targets, animal targets and human volunteers

Due to their small size and physical resemblance to physiological molecules such as proteins, NPs possess the capacity to revolutionise medical imaging, diagnostics, therapeutics as well as carry out functional biological processes. But these features may underlie their toxicity. Also, depending on the mode of administration and sites of deposition, toxicity may vary in severity. Therefore, to maintain clinical relevance, information on toxicity is presented using a system-based approach focusing on experimental lung, dermal, liver and brain targets (see Table 1).

## Lung targets

The lung is an attractive target for drug delivery due to the non-invasive nature of inhalation therapy, the lung's large surface area, localization/accumulation of drugs within the pulmonary tissue and avoidance of first-pass metabolism, thus reducing systemic side effects [82,83]. Nanocarrier systems for pulmonary drug delivery have several advantages which can be exploited for therapeutic reasons and, thus, are intensively studied.

## Polymeric nanoparticles

Polymeric NPs are biocompatible, surface modifiable and are capable of sustained drug release. They show potential for applications in the treatment of various pulmonary conditions such as asthma, chronic obstructive pulmonary disease (COPD), tuberculosis (TB) and lung cancer as well as extra-pulmonary conditions such as diabetes [84–89]. Already, there is a multitude of organic nano-polymers including collagen, gelatin, chitosan, alginate and bovine serum albumin (BSA). Furthermore, the last three decades has seen a rise in the development of synthetic polymers such as the biocompatible and biodegradable poly(lactic-*co*-glycolic acid) (PLGA) for use as drug carrier devices [90,91].

While such drug loaded nano-configurations demonstrate promising alternatives to current cancer treatment, cytotoxicity needs to be evaluated. PLGA NP successfully improve therapeutic outcome and reduce adverse effects via sustained and targeted drug delivery. Additionally, the use of biological capping materials such as chitosan or BSA further reduce toxicity while their biocompatibility and biodegradative capacity making them an intuitive choice for nanoparticulate surface modification. Romero et al. demonstrated a reduction in cytotoxicity of PLGA NPs stabilized with BSA compared to synthetic coating materials in cultured lung cancer cells [90]. Albumin, the most abundant serum protein, was found to be highly biocompatible making it a useful stabilizer for drug delivery vehicles. Similarly, chitosan-stabilization resulted in near-total cellular preservation and improved pulmonary mucoadhesion in an *in vivo* lung cancer model [31].

Biological capping materials reduce cytotoxicity by mimicking the physiological environment, thus ‘hiding’ from the immune system. However, the possibility of enzymatic degradation due to biophysical resemblance needs further investigation.

## Carbon nanotubes (CNTs)

CNTs are frequently used for *in vivo* inhalation models and can be subdivided into SWCNTs and MWCNTs with the former being considered more cytotoxic. This difference in toxicity has been attributed to the larger surface area of SWCNTs compared to the multi-layered alternative [92]. CNTs show biomedical potential in areas such as drug delivery, photodynamic therapy (PDT) and as tissue engineering scaffolds [93–95]. Previous studies have highlighted the toxic potential of both SWCNTs and MWCNTs; after murine intra-tracheal instillation of CNTs, pulmonary epitheloid granulomas and interstitial inflammation with subsequent fibrotic changes were observed [10,11,96]. Cytotoxicity is thought to be mediated by the up-regulation of inflammatory cytokines such as TNF-α. However, a recent study by Mutlu et al. postulates that toxicity after murine intra-tracheal instillation of SWCNTs arises due to nanotubular aggregation rather than the large aspect ratio of the individual nanotube [9]. This has led to the development of several methods to achieve improved nanotube dispersion [8]. Exposure of animals to MWCNTs results in contradictory reports with some authors appending toxicities in the range of asbestos poisoning whereas other studies found MWCNTs to be biocompatible and far from cytotoxic [35,96]. Such discrepancies may be explained by subtle variations in nanotube composition and ways of administration (intratracheal instillation versus whole-body inhalation) and warrants further investigation.

## Silica (SiO_2_) nanoparticles

Silica NPs are already in wide-spread use in the non-medical field as additives to chemical polishing, cosmetics, varnishes and food stuffs [36,97]. Relatively recently, such particles have been introduced into the biomedical field as biomarkers [98], cancer therapeutics [99] and drug delivery vehicles [100]. Silica NPs are considered ‘safe’ in moderate dosage (<20 μg/mL) as opposed to their crystalline pendants which are classed as class 1 carcinogens [36,37,101]. However, at a dose of 25 μg/mL silica NPs exhibited agglomerative potential *in vitro* and dose-dependent cytotoxicity at a critical concentration of 50 μg/mL as demonstrated on A549 cells [38,39]. Cell death was mediated by reactive oxygen species (ROS) induction and membrane lipid peroxidation. Apart from concentration dependence, silica NPs further exhibited particle size-dependent cellular toxicity with smaller diameters causing more harm than bigger ones as shown by Napierska et al. [40]. Paradoxical results have been obtained in animal studies focusing on the exposure of lungs to silica NPs. Previous studies on the pulmonary toxicity after intra-tracheal exposure of silica NPs have revealed profound acute pulmonary inflammation and neutrophil infiltration of lung tissue with the development of chronic granulomatous changes after 14 weeks in a dose-dependent manner [102]. However, subsequent longer-term studies utilizing the same NPs showed the induction of anti-inflammatory mediators and the reversibility of inflammatory and fibrotic changes to levels close to the control [103,104]. Fibrogenic mediators (IL-4, IL-10 and IL-13) were upregulated shortly after exposure to silica NPs and contributed to fibrotic changes [104]. These were counteracted by the overexpression of matrix-metalloproteinases (MMP), particularly MMP-2 and interferon gamma (INF-γ). Further to the expression of anti-fibrotic mediators, eventual recovery of lung tissue may be associated with the time-dependent reduction of NP concentration in the alveoli. Some studies suspect diffusion and translocation of NPs away from the lung tissue via the systemic circulation and deposition in extra-pulmonary organs [41,105,106]. The above suggests that it is theoretically feasible and within acceptable safety limits to use moderate doses of silica NPs; however high-dose toxicity profiles warrant further investigations.

## Silver nanoparticles

The most common route of pulmonary exposure to silver NPs (AgNP) is via the occupational inhalation of airborne particles during manufacturing [107]. Oberdorster et al. have shown in a rat model that inhaled NPs can translocate from their original site of deposition (e.g. lungs) to other tissues [105]. The current American Conference of Governmental Industrial Hygienist's (ACGIH) limit for silver dust exposure is 100 μg/m^3^. In order to evaluate potentially acute and delayed adverse pulmonary effects of AgNP, Sung et al. have carried out a series of inhalation studies focusing on the acute, subacute (28 days) and subchronic (90 days) toxicity of AgNP in rats [42,43,108]. In the acute setting, rats were exposed to different particle concentrations in a whole-body inhalation chamber for 4 consecutive hours and were subsequently observed for a further 2 weeks. At the highest concentration used (750 μg/m^3^; 7.5 times higher than the limit), no significant body weight changes or clinical changes were observed. Furthermore, lung function tests revealed no statistical differences between exposed and control groups. Repeated administration of AgNP for 4 weeks showed similar results. In contrast, subchronic inhalation for 13 weeks at a maximum concentration of 515 μg/m^3^ (5 times the limit) revealed time- and dose-dependent alveolar inflammatory and granulomatous changes as well as decreased lung function [44]. Such results suggest that while high-dose chronic exposure to AgNP has the potential to cause harm, under current guidelines and limits such excessive particle inhalation would seem unrealistic.

## Dermal targets

The skin is the largest organ of the body and functions as the first-line barrier between the external environment and the internal organs of the human body. Consequently, it is exposed to a plethora of non-specific environmental assaults within the air as well as to distinct and potentially toxic substances within creams, sprays or clothing. Topically applied NPs can potentially penetrate the skin and access the systemic circulation and exert adverse effects on a systemic scale.

## Silver nanoparticles

Ag is one of the most consistently studied NPs in terms of toxicity. This arises from the fact that AgNP possess proven anti-microbial effects which are currently used in many products ranging from wound dressings to clothing. However, the specificity of AgNP toxicity towards micro-organisms must be elucidated in order to exclude potentially adverse effects mediated by such particles on other exposed cell types within the body. Ag ingestion and topical application can induce the benign condition known as argyria, a grey–blue discoloration of the skin and liver caused by deposition of Ag particles in the basal laminae of such tissues [109]. This has prompted a limitation on the recommended daily dosage of Ag. Furthermore, numerous toxicity studies focusing on AgNP have been carried out on cell lines including mouse fibroblast, rat liver, human hepatocellular carcinoma and human skin carcinoma cells [5,45,46,110]. All observed a rise in ROS and oxidative-stress mediated cell death and apoptosis (concentrations between 2.5 and 200 μg/mL). The degree of toxicity was concentration-dependent and varied with surface coatings. Samberg et al. reported significant toxicity of uncoated AgNP on human epidermal keratinocytes in contrast to carbon-coated particles [47]. The exact mechanism of AgNP toxicity is unknown but ROS generation and oxidative stress are two likely routes. Once excessive ROS production outstrips the anti-oxidative capacity of the cell, oxidative stress is induced with subsequent production of inflammatory mediators, DNA damage and apoptosis [111]. Dermal penetration studies are ideally carried out on porcine skin due to its resemblance to that of humans in terms of thickness and rate of absorption [112]. Samberg et al. demonstrated porcine dermal biocompatibility after daily topical application of AgNP-containing cream over a period of 14 consecutive days (0.34–34 μg/mL). However, microscopically, dose-dependent oedema formation and hyperplasia were observed with particle deposition in superficial layers of the stratum corneum only. Trop et al. observed reversible silver toxicity in a human burns patient who was treated with a AgNP-coated dressing [48]. These results suggest reversible toxicity of AgNP and a transient discoloration of exposed skin.

## Titanium dioxide (TiO_2_) nanoparticles

TiO_2_ NPs have several properties which make them an advantageous ingredient for commercial sunscreens and cosmetics. They exhibit UV-light blocking properties and confer better transparency and aesthetics to creams. *In vitro* studies demonstrated cell type-dependent TiO_2_ toxicity affecting cellular functions such as cell proliferation, differentiation, mobility and apoptosis [49,113]. Such adverse effects, however, could not be replicated *in vivo*. In order to assess penetrative capacities, dermal infiltration studies have been carried out on human volunteers using different investigative techniques. Lademan et al. investigated the penetrative effect of repeated administration of TiO_2_-containing sunscreen on the skin of volunteers [50]. Tape stripping and histological appraisal of skin biopsies revealed that TiO_2_ penetrated into the open part of a hair follicle as opposed to the viable layers of the epidermis or dermis. Furthermore, the titanium amount in any given follicle was less than 1% of the applied total amount of sunscreen. Surface penetration via hair follicles or pores was also suggested by a study conducted by Bennat and Muller-Goymann where skin permeation was greater when sunscreen was applied to relatively hairy skins [51]. Mavon et al. demonstrated near total recovery of sunscreen after 15 tape strippings with no TiO_2_ deposition in hair follicles or skin layers [52]. It could be argued that different degrees of permeation and toxicity correlate with surface coatings and functionalizations of TiO_2_ NPs as well as with the number of follicular pores within the skin facilitating particle uptake.

## Silica nanoparticles

Silica NPs are frequently incorporated in drug additives and cosmetics as well as being used as nano-vehicles for drug delivery [114,115]. However, sparse literature on its cutaneous toxicity is available. Cell-based toxicity studies revealed a size-related increase in cytotoxicity when murine epidermal Langerhans cells were exposed to silica particles of diameters 70, 300 and 1000 nm [53]. Cellular uptake was more efficient for smaller particles (<100 nm) which correlated with an increased cytotoxicity. Park et al. evaluated toxicity of differently sized silica NPs on cultured human keratinocytes (CHK) and compared results with a human skin equivalent model (HSEM) as well as with an *in vivo* rabbit model [54]. Silica NPs exhibited significant dose-dependent toxicity on human keratinocytes with a statistically significant reduction in cell viability at a concentration of 50 μg/mL. A size-dependent increase in toxicity, as suggested by Jiang et al. could, however, not be confirmed [116]. Exposure of NPs to HSEM showed no inflammatory changes even at a maximum concentration of 500 μg/mL. Such low acute toxicity was confirmed with the *in vivo* rabbit skin model. These results highlight the superiority of HSEM compared to culture-based systems in terms of evaluating relative dermal toxicities of NPs and emphasize the discrepancies encountered if one tries to extrapolate results gained from cell-based studies into human scenarios.

## Gold nanoparticles

Due to facile means of synthesis and the potential for bio-functionalization, gold NPs (AuNP) are being investigated for clinical applications including dermal drug-delivery [117]. Sonavane et al. demonstrated size-dependent permeation on excised rat skin after topical application of differently sized AuNP (15, 102 and 198 nm) [58]. Smaller NPs penetrated deeper into the tissue than larger ones which were mainly accumulated in the more superficial epidermis and dermis. These findings may have important implications with regards to efficient NP-based dermal drug delivery. Au compounds are generally considered safe and have been in routine clinical use for many years, e.g. in the treatment of rheumatoid arthritis [118]. However, once reduced to nanometer scale, particles are known to undergo profound changes in terms of their biochemical properties which necessitates renewed investigations into their cytotoxic profile. Despite the relative wealth of toxicity studies focusing on AuNP, contradictory results remain the main obstacle to transition into the clinical setting. Several studies have demonstrated cellular uptake of AuNP to be a function of time, particle size and concentration. In a study by Mironava et al., human dermal fibroblasts were exposed to AuNP for a period of up to 6 days [55]. Three sets of NP concentrations were obtained for each of two different sizes. Larger particles, 45 nm, exhibited marked cytotoxicity at a concentration of 10 μg/mL compared to smaller particles, 13 nm in size, which only displayed cytotoxic signs at the much higher concentration of 75 μg/mL. These results conflict with those obtained by Pan et al. who reported maximum toxicity for a particle size of 1.4 nm [56]. Such differences may be explained by the distribution pattern of particles within cells and require more research.

## Liver targets

Being the site for first-pass metabolism, the liver is particularly vulnerable to NP toxicity and has consistently been shown to accumulate administered substances, even long after cessation of exposure. Thorough evaluation of NP-mediated hepatocellular toxicity thus remains of prevailing importance.

## Gold nanoparticles

AuNP play an interesting role in biomedicine and have multiple possible applications in the fields of imaging, drug and gene delivery [14,119]. A study conducted by Chen et al. revealed size-associated toxicity and lethality of AuNP [59]. Mice were exposed to intraperitoneal injection with naked AuNP ranging from 3 to 100 nm in size at a concentration of 8 mg/kg/week for 4 weeks. Particles sized 3, 5, 50 and 100 nm did not induce significant toxicity. However, particles ranging from 8 to 37 nm induced severe systemic adverse side effects in the test subjects such as fatigue, loss of appetite, weight loss and fur color changes. Death rates of mice exposed to that particular size range were near total. Histological examination of organs indicated Kupffer cell activation in the liver, splenic white pulp diffusion and structural deformities in lung parenchyma which were consistently associated with gold deposition at these sites. Toxicity was improved by surface modification of particles with a highly immunogenic peptide which induced an increased antibody response in the host. Despite improved cytocompatibility conferred by biological surface coatings, the primary mechanism of liver toxicity is thought to arise from acute inflammatory changes and subsequent apoptosis [60,61]. Increasingly, evidence shows that AuNP toxicity depends not only on the conventionally listed properties including surface functionalization but also on the route of administration [62,120]. It has been shown that intraperitoneal administration of particles is related to a significantly higher incidence of adverse effects than comparable intravenously delivered ones [62]. Despite the fact that these findings may have implications for clinical uses of such particles, caution should be exercised when stating that one route of administration gives a higher incidence of adverse effects than another, especially when based on toxicity results from different studies.

## Silver nanoparticles

Well known for their anti-microbial effects, AgNP may gain access into the circulation via various routes. Once within the systemic circulation, first-pass metabolism via the liver is likely, if not probable, thus potentially rendering hepatocytes vulnerable to toxic insults. Many studies have indicated the propensity for AgNP to accumulate within the liver and induce oxidative stress-related toxicity [63]. Certain parameters influencing the degree of toxicity include particle concentration [121], size [64], shape [65] and the ability to deplete cells of anti-oxidants [122]. Teodoro et al. demonstrated significant toxicity exerted by AgNP on BRL3A rat liver cells by measuring a significant reduction in mitochondrial function, a concomitant rise in lactate dehydrogenase (LDH) leakage from cells, significantly depleted levels of the antioxidant glutathione (GSH) as well as a rise in ROS concentrations [122]. Oxidative stress-dependent cytotoxicity was also confirmed by Kim et al. who were able to significantly improve viability of rat hepatoma cells exposed to AgNP after pre-treating cells with the naturally occurring anti-oxidant N-acetylcysteine [121].

## Silica nanoparticles

Biomedical applications for silica NPs are widespread and include diagnosis and drug delivery [123,124]. Despite such promising uses in the medical arena, a thorough evaluation of cytotoxicity is still lacking. Few studies have been carried out and contradictory results regarding toxicity further necessitate systematic research. Xie et al. demonstrated the hepatotoxic potential of silica NPs causing mononuclear inflammatory cell infiltrates at the portal area with concomitant hepatocyte necrosis [66]. Such inflammatory changes are postulated to relate to frustrated phagocytosis of larger silica NPs (>100 nm) with subsequent stimulation of pro-inflammatory cytokine release [67]. A size relationship has also been reported by Kumar et al. who found small silica NPs (<25 nm) to be biocompatible and non-toxic to murine liver parenchyma [68]. In contrast to these findings, Nishimori et al. suggests acute toxicity related to small silica NPs (<100 nm) [69]. Several factors may lead to these apparently inconsistent results; the presence or absence and type of coating is one of the major parameters in determining interactions between the NPs and physiological environment as is the particle's size. However, contradictory results in terms of size-dependent toxicity warrant further investigations into the role of different routes of particle administration. The results for silica NPs suggest that less invasive means of administration (e.g. oral) reduce organ-specific toxicity compared to intravenously dispensed particles. Building on this theory, intravenously given larger NPs (>100 nm) may prove less cytotoxic due to impaired extravasation through minute capillary fenestrae and thus, little organ-specific deposition, as postulated by Nishimori et al. [69].

## Quantum dots

Semiconductor nanocrystals, or QDs may be used in a variety of biomedical applications. The general structure of QDs comprises an inorganic core–shell and an organic coating to which biomolecules may be conjugated to enable targeting to specific areas within the body. Such close proximity and interaction with the physiological environment necessitates toxicological evaluation of these particles. Cell based studies focusing on QD-induced adverse effects found toxicity most likely arises from the liberation of metal ions released from the heavy metal core [70]. Oxidative environments further promote degradation and metal ion leaching. The liver is of particular importance with regards to bio-toxicity because of first-pass metabolism and potential accumulation and deposition within the organ, as shown by Yang et al. [125]. QD size was also postulated to be a major parameter in organ-specific deposition with smaller particles (<20 nm) extravasating through capillary fenestrae that are large enough in the liver (∼100 nm in size) [71]. The long half-life clearly has implications for organ toxicity, particularly in view of the liver's untoward propensity to heavy metal ion poisoning which makes exposure to QDs potentially very hazardous. Surface coating to protect the core from degradation has been shown to reduce toxicity [126]. Conventionally, QDs are coated with a layer of zinc sulphide (ZnS) or mercaptoacetic acid. However, evidence of continued cellular toxicity after prolonged periods of time suggests either inadequate core coverage or the need for a different type of coating material [72]. Clift et al. carried out a series of experiments assessing additional surface coatings for their respective cytotoxicities [73]. CdTe/CdSe cored QDs with a ZnS shell were additionally covered with organic, carboxylated (COOH), amino (NH_2_) or poly(ethylene glycol) (PEG) coatings. Cytotoxicity was tested on exposure to each type separately by measurement of macrophage cell viability and LDH release. All QDs were shown to induce significant cytotoxicity after 48 h and coating materials as well as liberated Cd ions were suggested to be the causative agents. It is likely that a breakdown of physically labile surface material resulted in ion liberation and subsequent toxicity. Recently, Seifalian and colleagues have demonstrated that the novel synthetic nanomaterial polymeric oligohedral silsesquioxane (POSS), when incorporated onto CdTe cored QDs, shows significantly enhanced cytocompatibility than conventionally used materials, even without ZnS shelling (unpublished data). POSS was shown to be non-toxic by preventing ion leakage from the core. These results underline the importance of the type of coating material used and suggest that the most important factor influencing QD toxicity remains heavy metal ion leakage from the core due to inadequate surface coverage.

## Brain targets

The brain, unlike the liver, has very limited regenerative capacity and must therefore be particularly protected from exogenous insults. This is achieved by the protective BBB which separates the cerebrospinal fluid (CSF) surrounding the brain from the systemic blood circulation via tight junctions around the capillaries and is of undoubted benefit in preventing blood-borne pathogens from gaining entry and causing potentially irreversible damage while allowing the diffusion of smaller lipophilic molecules such as oxygen. However, bypassing the BBB may be beneficial and potentially life-saving in the management of acute conditions such as cerebral meningitis as well as chronic illnesses like dementia or Parkinson's disease. Targeted drug delivery allows the use of smaller drug doses and hence reduces systemic adverse effects. Nevertheless, the fate of NPs after translocation into the structures of the central nervous system (CNS) requires toxicological analyses. External insult to the BBB could potentially activate cerebral epithelial cells thus inducing oxidative stress-mediated injury. Additionally, the integrity and biostability of the particle's coating materials require rigorous assessment [127].

Recently, Oberdorster et al. demonstrated the uptake of NPs by sensory nerve endings within the pulmonary epithelium and subsequent axonal translocation into the CNS which presents a further route of exposure to potentially harmful NPs [127].

## Gold nanoparticles

AuNP are valuable imaging modalities for *in vitro* and *in vivo* cell tracking. Studies suggesting AuNP as imaging agents of the CNS are manifold and could prove to be useful in the diagnoses and therapeutics of CNS pathologies [128–130]. However, such enthusiasm is dampened by the relative paucity of data on the interactive capabilities of AuNP with the cells of the CNS. Primary brain microvascular endothelial cells (BMECs) are frequently used to provide information on BBB transport characteristics and molecular mechanisms and offer an opportunity to investigate into the interactions between NPs and the surface of the BBB [131–133]. It has been postulated that the release of pro-inflammatory cytokines (e.g. TNF-α, IL-β and IL-2) induces an increase in permeability and toxicity in rat brain microvessel endothelial cells (BMECs) exposed to AgNP which seemed both size- and time-dependent [76]. In contrast, a similar study utilizing AuNP of comparable dimensions did not evoke increased secretion of inflammatory markers nor was enhanced cellular permeability detectable [74]. These findings are supported by a study carried out on RAW264.7 macrophage cells which demonstrated no pro-inflammatory cytokine release in response to AuNP exposure [75]. However, mild toxicity was observed with smaller AuNP (3 nm) compared to larger ones (5 nm). Such size-dependent toxicity has previously been observed in various cell lines [134,135] and *in vivo* models [136,137]. Ultra-small NPs were found to be widely distributed to almost all tissues including the brain whereas larger ones were barred access to cerebral tissues. This could be due to either the physical hindrance provided by the BBB or the faster and more efficient removal of larger particles from the circulation compared with smaller ones. Consequently, a lower particle concentration would reach and subsequently be absorbed by the BBB. The BBB's regulatory function as well as the removal-theory were further demonstrated in a different study where, even after repeated administration of relatively large AuNP (12.5 nm), the amount in the brain was significantly lower than that in the liver, spleen and kidneys [138].

## Silver nanoparticles

In contrast to Au, AgNP have been shown to exhibit neurodegenerative abilities [139,140]. Recent research focusing on the interaction between AgNP and BBB has revealed functional disruption of the BBB and subsequent brain oedema formation [139]. In support of these findings, Tang et al. demonstrated AgNP-induced BBB destruction, astrocyte swelling and neuronal degeneration in a rat model [140,141]. The pattern of distribution and degree of toxicity was found to be concentration- and size-dependent [64]. A study conducted by Costa et al. suggests mitochondrial dysfunction and impaired energy production as the possible underlying mechanism of neurodegeneration [77]. Repeated administration resulted in significant accumulation in organs with smaller AgNP (<100 nm) being predominantly picked up by the liver while larger particles (>100 nm) mainly accumulated in the spleen. This pattern of particle uptake is regulated by the pore size of capillary fenestrae (∼100 nm). Dose-dependent accumulation has previously been observed in rats following repeated oral doses of AgNP [78]. Human subjects demonstrated a greyish hyperpigmentation of their skin following oral ingestion of AgNP [142,143]. These results suggest the capacity for gastrointestinal absorption of AgNP with subsequent systemic distribution and accumulation in various tissues.

This apparent potential for AgNP to accumulate and cause harm requires further investigation with particular attention assigned to the influence of NP size on the distribution and toxicity. Powers et al. demonstrated the potential for AgNP to behave as a developmental neurotoxin in zebrafish embryos and cause long-term changes in synaptic functioning [79]. Exposure to ionized silver (Ag^+^) resulted in a marked increase in turnover of the neurotransmitters dopamine (DA) and serotonin (5-HT) which are key components in the reward, anxiety and sensorimotor pathways. Ag^+^-induced neurotransmitter hyperactivity resulted in behavioural changes consistent with a lower anxiety-threshold.

## Superparamagnetic nanoparticles

SPIONs and ultra-small SPIO nanoparticles (USPIONs) consist of an iron oxide core and a variable carbohydrate coating which determines cellular uptake and biological half life. The degree of surface coverage has been postulated to be the main parameter in cellular uptake as incomplete surface coverage was shown to promote opsonization and rapid endocytosis whereas fully coated SPION escaped opsonization which, as a result, prolonged plasma half-life [144]. However, more recently, particle size as opposed to coating degree has been suggested to exert chief influence on the rates of uptake by macrophages [145]. Being one of the few FDA approved NPs for the use in MRI, SPION most commonly find applications in the imaging of the vasculature and lymph nodes [146–149]. However, recent reports from both animal models and human subjects have shown their efficacy in visualizing intracerebral malignancies and neurological lesions within the CNS [150,151]. Despite such routine use of SPION, the long-term effects and potential neurotoxicity have, as yet, not been evaluated extensively.

The unique physio-chemical properties shared by all NPs, such as nanometer size and a large surface area to volume ratio, makes SPION particularly valuable for novel therapeutic and diagnostic applications. However, such dimensional reductions may potentially induce cytotoxicity and interfere with the normal components and functions of the cell [152,153]. Previous *in vitro* studies have shown the capacity for SPION to induce ROS generation, impair mitochondrial function and cause leakage of LDH – all of which could incite neurotoxicity as well as potentially aggravate pre-existing neuronal damage [154–156]. Furthermore, toxicity reports demonstrated an association between particle size, type of surface coating and breakdown products, concentration and the degree of opsonization and cytotoxicity in cultured cells [157–159]. For example, Berry et al. utilized fibroblast cultures to demonstrate the ability to tune particle toxicity according to particle coating. They compared the *in vitro* toxicity of plain, uncoated magnetic iron oxide NPs (P particles) with either dextran derivatized (DD) or albumin derivatized (AD) NPs. P particles as well as DD particles exhibited similar toxicities, whereas AD particles managed to induce cell proliferation [157].

In a study by Muldoon et al., the distribution, cellular uptake and toxicity of three FDA approved SPION of different sizes and surface coatings were compared to each other and to a laboratory reagent [80]. Firstly, inoculation of ferumoxtran-10 (USPION: 20–50 nm in size, complete surface coverage with native dextran), ferumoxytol (USPION: 20–50 nm in size, complete surface coverage with semisynthetic carbohydrate) and ferumoxide (SPION: 60–185 nm in size, incompletely coated with dextran) as well as the lab reagent MION-46 into tumor-bearing rat brains demonstrated direct uptake of ferumoxtran-10 into tumor tissue and long-term retention within the cancerous lesion (5 days). However, uptake seemed NP dependent. Ferumoxide inoculation did not yield tumor enhancement which suggests size and surface coverage dependence. The second step involving osmotic BBB disruption to evaluate transvascular SPION delivery and neurotoxicity displayed no evidence of gross pathology implying the feasibility of intracerebral injection of clinical USPION into humans.

## Quantum dots

QDs may be functionalized to serve both diagnostic and therapeutic purposes. Presently, QDs find invaluable experimental applications in visualizing neural stem and progenitor cells in developing mouse embryos *in vivo* [160], act as nano-vehicles for brain-targeted drug delivery [161] and demarcate brain malignancies for precise tumor resection [162]. Previously, it has been suggested that NPs can bypass the BBB and reach the brain via the olfactory epithelium or trigeminal nerve to potentially cause harm [163]. In order to investigate the potential to reach the structures of the CNS, Kato et al. intraperitoneally injected CdSe/ZnS-core/shell-typed QDs functionalized with captopril with a diameter of 13.5 nm into mice [81]. Six hours post-injection, organs were harvested and brains were dissected into several areas including the olfactory bulb, cerebral cortex, hippocampus, thalamus and brainstem. Despite the detection of unexpectedly high amounts of Cd within the brain tissues, no signs of inflammation, neuronal loss or functional disorders were present. Several possible routes of entry into the brain have been suggested. Small QDs may transfer through minute gaps (<20 nm) between astrocytic foot processes which constitute the BBB out of the capillary bed into the brain parenchyma. Alternatively, interaction between QD-cap and the receptors located at the BBB may lead to the uptake of QDs via phagocytosis or pinocytosis [164]. However, no evidence for the presence of captopril receptors at the BBB has been published making receptor-mediated uptake unlikely. Another postulated route of entry into the brain is the retrograde axonal transport from gut neurones via peripheral nerves into the tissues of the CNS. However, according to the published report, no QD-cap was found in peripheral nerves. These results suggest that for clinical or therapeutic purposes, the route across the BBB may show promise in the future management of CNS pathologies. However, the combination of scarce clinical data on the toxic effects with the lack of long-term *in vivo* toxicity studies calls for the need for considerable attention to the systematic evaluation of QDs intended for brain-specific purposes.

## Conclusion

NPs have certain unique characteristics which can be and have been exploited in many biomedical applications. However, these unique features are postulated to be the grounds for NP-induced biotoxicity which arises from the complex interplay between particle characteristics (e.g. size, shape, surface chemistry and charge), administered dose and host immunological integrity. Recently, more emphasis has been placed onto understanding the role of the route of particle administration as a potential source for toxicity. Current research focuses on elucidating the mechanistics underlying NP toxicity which are postulated to range from inflammatory cell infiltration and cellular necrosis to ROS-induced apoptosis. Despite the wealth of toxicity studies available, the authors have identified several points of criticism which currently hinder the progression into clinical settings. Firstly, the application of the so-called ‘proof of principle’ approach where cell cultures or experimental animals are exposed to ultra-high NP concentrations to ensure cytotoxicity leads to unrealistic results which cannot be extrapolated into the human scenario since diagnostic and therapeutic interventions usually only require the administration of minimal concentrations. Thus, not only are we faced with scientifically unreliable data, such practices may, quite dangerously cause unnecessary alarm in the public. Additionally, the authors have identified two further limitations of current toxicity studies; firstly, the chronicity of NPs exposure in the case of therapeutic applications needs more thorough long-term evaluation; secondly, different studies apply different particle formulations leading to conflicting and unreliable results. Consequently and for the future, more emphasis should be placed on defining the dose of NPs in relation to the route of administration. As mentioned at the beginning, end-organ accumulation and distribution as well as metabolism and excretion are variable depending on the routes of administration, such that intravenous NP administration may have more implications with regards to systemic adverse effects than dermal application of NPs. However, one must treat and compare toxicity results from different studies with caution, as current toxicity protocols lack uniformity with respect to NP formulations and application protocols. It has become apparent that a unifying protocol for the toxicological profiling of NPs may be required in order to achieve reliable outcomes that have realistic implications for the human usage of NPs. In summary, current difficulties in evaluating NP toxicity originate in the inherent discrepancies found amongst toxicity study protocols such that is has become apparent that a unifying protocol for the toxicological profiling of NPs may be required in order to achieve reliable outcomes that have realistic implications for the human usage of NPs. Not only is there a pressing need for long-term studies, the future of nanotoxicology must also more heavily rely on realistic particle dosages and composition principles as well as differentiating more carefully between various routes of administrations if nanotechnology is to fully unfold its clinical potential.

## Figures and Tables

**Table 1:** Summary of *in vitro* and *in vivo* evaluations of nanoparticle toxicity.

Target	Nanoparticle	Conjugation	Concentration (time/size)/route of administration	Cellular target	Animal target	Major outcomes	Ref.
Lung	PLGA NP	Chitosan	300-5000 μg/mL (4 h)	A549 human lung cancer cells		Non-toxic even at highest concentrations.	[31]
	Solid lipid NP		500 μg/mL (24 h)	A549 human lung cancer cells		No inflammatory changes in lung parenchyma at the critical concentration of 500 μg/mL. Concentrations lower than 200 μg/mL are thought to be safe.	[32]
	SWCNT		1.56-800 μg/mL (24 h)	A549 human lung cancer cells		Low acute cytotoxicity was further reduced by dispersion of SWCNTs in serum.	[33]
	SWCNT		1 or 5 mg/kg (24 h, 1 week, 1 month, 3 months) Intratracheal instillation		Sprague-Dawley rats	Mortality in 15% of animals after 24 h exposure to highest dose due to physical blockage of airways rather than acute inflammation. Multifocal granulomatous change on histology - apparently no relation to dose or time. No inflammatory change.	[10]
	SWCNT		1.5 mg/kg (30 days) Intratracheal instillation		C57Bl/6 mice	Significant reduction in inflammatory and fibrotic changes after exposure of serum-dispersed particles relative to the non-dispersed pendent. Toxicity is attributable to particle aggregation rather than physiochemical property of individual nanotube.	[9]
	SWCNT	PEG	47 mg on days 0 and 7 (follow-up: 4 months) Intravenous infusion		Nude mice	No significant inflammatory changes were observed, however, particle deposition in liver macrophages was observed.	[34]
	MWCNT		0.5, 2 or 5 mg/animal (3 and 15 days) Intratracheal instillation		Sprague-Dawley rats	Dose-dependent increase in inflammatory markers post-BAL. Dose-dependent fibrotic change and interstitial granuloma formation.	[11]
	MWCNT		0.2, 0.5 or 2.7 mg/kg (7, 14 days) Inhalation		C57BL/6 mice	Uniform particle uptake by pulmonary macrophages. No inflammatory or fibrotic changes were observed.	[35]
	Silica NP		10-100 μg/mL (24 h, 48 h and 72 h)	A549 human lung cancer cells		Dose- and time-dependent decrease in cell viability: up to 50% reduction at highest dosage after 72 h. Oxidative stress indicated as mechanism of cytotoxicity.	[36]
	Silica NP		5, 10, 20, 50 or 100 μg/mL (24 h)	Primary mouse embryo fibroblasts (BALB/3T3)		Dose-dependent reduction in cell viability. Excessive ROS generation and GSH depletion suggest oxidative cell damage as the underlying mechanism of cytotoxicity.	[37]
	Silica NP		25 μg/mL (24 h)	A549 human lung cancer cells HepG2 cells RPMI 2650 human nasal septal epithelial cells N2a mouse neuroblast cells		Nuclear protein aggregation and subsequent interference with gene expression resulting in inhibition of replication, transcription and cell proliferation.	[38]
	Silica NP		0-185 μg/mL (24 h)	A549 human lung cancer cells EAHY926 endothelial cells J774 monocyte macrophages		Dose-dependent increase in cytotoxicity.	[39]
	Silica NP		33-47 μg/cm^2^ (small NP), 89-254 μg/cm^2^ (larger NP) (24 h)	EAHY926 endothelial cells		Size-dependent reduction in viability with smaller particles in the nanoscale exhibiting higher toxicity compared to particles >100 nm.	[40]
	Silica NP		20 mg/animal (1 or 2 months) Intratracheal instillation		Wistar rats	Nano-sized silica particles produced relatively lower pulmonary fibrosis compared to micro-sized silica particles. This is thought to be due to the translocation of ultrafine nanosilica away from the lung parenchyma.	[41]
	Silver NP		750 μg/m^3^ (4 h for 2 weeks) Inhalation		Sprague-Dawley rats	No significant changes in lung function and body weight in exposed groups compared to fresh air controls.	[42]
	Silver NP		61 μg/m^3^ (6 h/day, 5 days/week for 4 weeks) Inhalation		Sprague-Dawley rats	No significant clinical changes or changes in haematology and blood biochemical values.	[43]
	Silver NP		515 μg/m^3^ (6 h/day, 5 days/week for 13 weeks) Inhalation		Sprague-Dawley rats	Dose- and time-dependent increase in blood Ag nanoparticle concentration was observed along with correlating increases in alveolar inflammation and small granulomatous lesions.	[44]

Dermal	Silver NP		50 and 100 μg/mL (24 h)	NIH3T3 (mouse fibroblasts)		Mitochondria-dependent cellular apoptosis associated with ROS at a concentration of ≥50 μg/mL.	[45]
	Silver NP		0.76-50 μg/mL (24 h)	A431 (human skin carcinoma)		No evidence for cellular damage up to a concentration of 6.25 μg/mL. Morphological changes at concentrations between 6.25 and 50 μg/mL with concomitant rise in GSH, SOD and lipid peroxidation. DNA fragmentation suggests cell death by apoptosis.	[46]
	Silver NP		0-1.7 μg/mL (24 h)	HEK cells		Significant dose-dependent decrease in cell viability at a critical concentration of 1.7 μg/mL with concomitant rise in inflammatory cytokines (IL-1β, IL-6, IL-8, and TNF-α).	[47]
			0.34-34.0 μg/mL (14 consecutive days)		Porcine skin	No gross irritations macroscopically. Ultrastructural observations revealed areas of focal inflammation and localization of Ag NPs in stratum corneum of the skin.	
	Silver NP		Silver-coated wound dressing ‘Acticoat’ (1 week)		Human burns patient	Reversible hepatotoxicity and argyria-like discoloration of treated area of skin, elevated plasma and urine silver concentrations and increased liver enzymes.	[48]
	TiO_2_ NP		15 μg/cm^2^ (24 h)	HaCaT (keratinocyte cell line), human dermal fibroblasts, human immortalized sebaceous gland cell line (SZ95)		Cytotoxicity was observed affecting cellular functions such as cell proliferation, differentiation and mobility resulting in apoptosis.	[49]
	TiO_2_ NP		2 mg/cm^2^ sunscreen applied to volar forearm 5× on days 1, 2 and 3; 1× on day 4 (tape stripping 1 h post repetitive application of sunscreen)		Human volunteers	Tape-stripping revealed no nanoparticles in the deeper layers of the stratum corneum. Small amounts of NP (<1% of total amount of sunscreen applied) could only be identified within pilosebaceous orifices.	[50]
	TiO_2_ NP		NP containing sunscreen		Human volunteers	Increased skin permeation of NP when sunscreen was applied at hairy skin of human volunteered.	[51]
	TiO_2_ NP		2 mg/cm^2^ sunscreen applied to external surface of upper arm (tape stripping 5 h post application of sunscreen)		Human volunteers	>90% of sunscreen recovered in first 15 tape strippings. Remaining 10% did not penetrate into viable tissue.	[52]
	Silica NP		70, 300 and 1000 nm in size	XS52 (murine Langerhans cells)		Size-related toxicity with faster cellular uptake of smaller particles and concomitant higher toxicity.	[53]
	Silica NP		30-300 μg/mL (48 h)	CHK (human keratinocytes)		Reduced cell viability.	[54]
			500 μg/mL (5 or 18 h)	HSEM		No irritation at 500 μg/mL.	
			500 μg/mL (24 and 72 h)		*In vivo* rabbit model (Draize skin irritation test)	No erythema or oedema formation observed - even on tape-untreated animals.	
	Gold NP		95, 142 and 190 μg/mL (13 nm) 13, 20 and 26 μg/mL (45 nm) (3 or 6 days)	CF-31 (human dermal fibroblasts)		Cytotoxicity was size- and dose-dependent. Larger particles (45 nm) exhibited greater toxicity at smaller doses (10 μg/mL) compared to smaller ones (13 nm) which only exhibited cytotoxicity at a concentration of 75 μg/mL.	[55]
	Gold NP		0.8-15 nm in size (48 h)	SK-Mel-28 (melanoma cells), L929 mouse fibroblasts		Maximum cytotoxicity with smaller NP (1.4 nm) characterized by apoptosis and necrosis.	[56]
	Gold NP	Citrate	0-0.8 μg/mL (14 nm in size) (2, 4 or 6 days)	Human dermal fibroblasts		Dose-dependent reduction in cell proliferation.	[57]
	Gold NP		15, 102 and 198 nm in size		Excised abdominal skin of Wistar rats	Size-dependent permeation through rat skin with smallest NP having deeper tissue penetration	[58]

Liver	Gold NP	Immunogenic peptides: • pFMDV • pH5N1	8 mg/kg/week (3-100 nm in size) (4 weeks) Intraperitoneal		BALB/C mice	Naked NP: severe adverse effects with resultant death with particles ranging from 8 to 37 nm in diameter. Microscopically, Kupffer cell activation in the liver and lung parenchymal destruction was observed. Surface modified NP: elicited increased host immune response and improved cytocompatibility.	[59]
	Gold NP	PEG	0.17, 0.85 and 4.26 mg/kg body weight (13 nm in size) (30 min after injection for 7 days) Intravenous		BALB/C mice	NPs were found to accumulate in liver and spleen. Significant upregulation of inflammatory cytokines (IL-1, 6, 10 and TNF-α) with subsequent apoptosis of hepatocytes at highest concentrations (4.26 mg/kg). No significant changes in the liver at lower doses.	[60]
	Gold NP	PEG	4.26 mg/kg (4 and 100 nm in size) (30 min) Intravenous		BALB/C mice	Both 4 and 100 nm sized gold NP upregulated genes responsible for inflammation, apoptosis and cell cycle.	[61]
	Gold NP		0.14-2.2 mg/kg (13.5 nm in size) (14-28 days) Per oral, intraperitoneal or intravenous			Highest toxicity was found with oral and i.p. administration whereas lowest toxicity was seen with tail vein injection.	[62]
	Silver NP		30 or 120 μg/mL dispersed in fish tank (24 h)		Zebrafish	Oxidative stress-mediated toxicity due to free Ag^+^ liberation. Induction of pro-apoptotic signals in liver tissues.	[63]
	Silver NP		23.8, 26.4 or 27.6 μg/mL single or repeated administration (20, 80 and 110 nm, respectively), once daily for 5 consecutive days (1, 3, 5 days) Intravenous		Wistar rats	Size-related tissue uptake with smaller NP (20 nm) showing higher concentrations in organs than larger ones. Accumulation of NP after repeated administration has implications for tissue toxicity.	[64]
	Silver NP		6.25-100 μg/mL for primary fibroblasts and 12.5-200 μg/mL for primary liver cells (7-20 nm sized spheres) (24 h)	Primary mouse fibroblasts, primary hepatocytes		NP enter cells which results in the production of mediators of oxidative-stress. However, protective mechanisms could be observed which increase GSH production to avoid oxidative damage.	[65]
	Silica NP		0.001 μg/mL (1, 3, 7, 15, and 30 days) Intravenous		ICR mice	Principle end-organs for NP accumulation were liver, spleen and lungs. Mononuclear cell infiltration at hepatic portal area and hepatocyte necrosis were observed.	[66]
	Silica NP		50 mg/kg (50, 100 or 200 nm in size) (12, 24, 48 and 72 h, 7 days) Intravenous		BALB/C mice	Size-dependent hepatic toxicity with inflammatory cell infiltrates. Macrophage-mediated frustrated phagocytosis of larger NP (100 and 200 nm) resulted in release of pro-inflammatory cytokines and cell infiltrates within hepatic parenchyma.	[67]
	Silica NP	PEG	2 mg/kg (20-25 nm in size) (24 h) Intravenous		Nude mice	Greatest accumulation of NP in liver, spleen and intestines but no pathological changes were observed with small NP (<25 nm). Near-total excretion of NP via the hepatobiliary system.	[68]
	Silica NP		10-100 mg/kg (70, 300 or 1000 nm in size) Intravenous		BALB/C mice	Significant hepatotoxicity (degenerative necrosis of hepatocytes) was observed with smaller NP (<100 nm) whereas no pathological changes were seen with larger particles (300 or 1000 nm), even at relatively higher concentrations of NP (100 mg/kg).	[69]
	CdSe QD	±ZnS shell	62.5, 250 and 1000 μg/mL (24 h)	Primary rat hepatocytes		Cytotoxicity was thought to be due to the release of free cadmium ions which could not be fully eliminated by ZnS coating of the OD core.	[70]
	CdSeTe OD	ZnS shell PEG	40 pmol (18.5 nm in size) (1, 4 and 24 h; 3, 7, 14 and 28 days) Intravenous		ICR mice	Extravasation of small QD (<20 nm) via hepatic capillary fenestrae (∼100 nm) and deposition within liver parenchyma.	[71]
	CdSe QD	ZnS shell	62.5, 100 and 250 μg/mL (24, 48 or 72 h)	HepG2 cells		Dose-dependent cytotoxicity. In extreme conditions (250 μg/mL for 72 h) a reduction in cell viability of almost 40% was observed which correlated with an increase in free cadmium ion concentration of 1.51 ppm.	[72]
	CdTe/CdSe QD	ZnS shell + either of the following: • organic coating • COOH • NH_2_ • PEG	20, 40 or 80 nM (2, 4, 24 and 48 h)	J774.A1 (murine ‘macrophage-like’ cells)		Regardless of coating, all QD induced significant cytotoxicity after 48 h as measured by cell viability and LDH release.	[73]

Brain	Gold NP		0.8-50 μg/mL (3, 5, 7, 10, 30 and 60 nm) (24 h)	rBMEC (primary rat brain microvessel endothelial cells)		No morphological changes could be detected after 24 h suggesting cytocompatibility of the NP tested. Only the smallest NP tested (3 nm) induced mild signs of cellular toxicity.	[74]
	Gold NP		(12.5 nm in size) (40, 200 or 400 μg/kg/day for 8 days) Intraperitoneal		C57/BL6 mice	Small amounts of NP were able to cross the BBB but did not induce evident neurotoxicity.	[75]
	Silver NP		6.25-50 μg/mL (25, 40 or 80 nm in size) (24 h)	rBMEC (primary rat brain microvessel endothelial cells)		Time- and dose-dependent increase in pro-inflammatory cytokine release and correlating increases in permeability and cytotoxicity of cells.	[76]
	Silver NP		10, 25 or 50 μg/mL (1 h)	Wistar rat tissue and homogenates		*In vitro* activities of mitochondrial respiratory chain complexes I, II, III, and IV were decreased in the brain and other tissues thus increasing the potential for oxidative stress-induced cell damage.	[77]
	Silver NP		30, 300 or 1000 mg/kg/day for 28 days (60 nm in size) Per oral		Sprague-Dawley rats	Dose-dependent accumulation of NP was observed in the brain and other organs suggesting systemic distribution after oral administration. ALP and cholesterol increased significantly in high-dose group (1000 mg/kg/day) indicating hepatotoxicity.	[78]
	Silver NP		0.03, 0.1 or 0.3 μM (4 h pf-5 days pf)		Zebrafish embryos	Neurobehavioural abnormalities were observed in adult zebrafish with increased DA and 5HT turnover in previously exposed embryos secondary to altered synaptic functioning.	[79]
	(U)SPION		208 or 1042 μg/mL of: • Ferumoxtran-10 (20-50 nm) • Ferumoxytol (20-50 nm) • Ferumoxide (60-185 nm) (3 months) Intracerebral inoculation or Intra-arterial after BBB disruption		Long Evans rats	Direct inoculation of all 3 SPION agents resulted in the uptake into the CNS parenchyma. No pathological changes were detected.	[80]
	CdSe QD	ZnS shell Captopril (cap) conjugation	0.68 mg containing 50 nmol Cd (13.5 nm in size) (6 h) Intraperitoneal		ICR mice	Relatively high amounts of Cd ions found in brain tissue but no signs of inflammation or parenchymal damage were observed.	[81]

*Key*: 5-HT, serotonin; Ag/Ag^+^, silver/silver ion; ALP, alkaline phosphatase; BAL, broncho-alveolar lavage; BBB, blood-brain-barrier; CdSe(Te), cadmium selenide (telluride); CNS, central nervous system; DA, dopamine; GSH, glutathione; i.p., intraperitoneal; LDH, lactate dehydrogenase; MMP, matrix metallo-proteinase; MWCNT, multi-walled carbon nanotube; NP, nanoparticle; PEG, poly(ethylene glycol); pf, post-fertilization; PLGA; poly(lactic-*co*-glycolic acid); ROS, reactive oxygen species; SWCNT, single-walled carbon nanotube; (U)SPION, (ultra) small iron oxide nanoparticle; and ZnS, zinc sulphide.
